# Spatiotemporal Distribution of Tuberculosis in the Oromia Region of Ethiopia: A Hotspot Analysis

**DOI:** 10.3390/tropicalmed8090437

**Published:** 2023-09-07

**Authors:** Dereje Bekele, Solomon Aragie, Kefyalew Addis Alene, Tariku Dejene, Samson Warkaye, Melat Mezemir, Dereje Abdena, Tesfaye Kebebew, Abera Botore, Geremew Mekonen, Gadissa Gutema, Boja Dufera, Kolato Gemede, Birhanu Kenate, Dabesa Gobena, Bizuneh Alemu, Dagnachew Hailemariam, Daba Muleta, Gilman Kit Hang Siu, Ketema Tafess

**Affiliations:** 1 Communicable and Non-Communicable Diseases Prevention and Control Directorate, Oromia Region Health Bureau, Addis Ababa P.O. Box 24341, Ethiopia; dkumsa2000@gmail.com (D.A.); geremew_mek@yahoo.com (G.M.); kgkolato05new@gmail.com (K.G.); wabizuneh@gmail.com (B.A.); dagneh78@yahoo.com (D.H.); 2 Department of Microbial, Cellular and Molecular Biology, College of Natural and Computational Sciences, Addis Ababa University, Addis Ababa P.O. Box 1176, Ethiopia; swondimkun@gmail.com (S.A.); gadissagutema@gmail.com (G.G.); duferataddese@yahoo.com (B.D.); 3 Geospatial and Tuberculosis Team, Telethon Kids Institute, Perth, WA 6009, Australia; kefyalew.alene@curtin.edu.au; 4 School of Public Health, Faculty of Public Health Sciences, Curtin University, Perth, WA 6102, Australia; 5 Center for Population Studies, College of Development Studies, Addis Ababa University, Addis Ababa P.O. Box 1176, Ethiopia; tarikud@gmail.com; 6 Ethiopian Public Health Institute, National Data Management Center for Health, Addis Ababa P.O. Box 1242, Ethiopia; samethiopia05@gmail.com; 7 Health Promotion and Diseases Prevention Directorate, Addis Ababa City Administration Health Bureau, Addis Ababa P.O. Box 30738, Ethiopia; melatmezemr@gmail.com; 8 Public Health Emergency Management, Research, and Blood Bank Service Directorate, Oromia Region Health Bureau, Addis Ababa P.O. Box 24341, Ethiopia; tesfayekebebew2003@gmail.com (T.K.); aberabotore22@gmail.com (A.B.); simboyt@gmail.com (B.K.); dabesagobena25@gmail.com (D.G.); dabamulleta@gmail.com (D.M.); 9 National HIV/AIDS and TB Research Directorate, Ethiopian Public Health Institute, Addis Ababa P.O. Box 1242, Ethiopia; 10 Bacterial, Parasitic, and Zoonotic Research Directorate, Ethiopian Public Health Institute, Addis Ababa P.O. Box 1242, Ethiopia; 11 Department of Health Technology and Informatics, The Hong Kong Polytechnic University, Hung Hom, Hong Kong; gilman.siu@polyu.edu.hk; 12 Department of Applied Biology, School of Applied Natural Science, Adama Science and Technology University, Adama P.O. Box 1888, Ethiopia; ttafess@gmail.com; 13 Institute of Pharmaceutical Science, Adama Science and Technology University, Adama P.O. Box 1888, Ethiopia

**Keywords:** tuberculosis, TB case notification, spatiotemporal, Oromia, Ethiopia

## Abstract

Tuberculosis (TB) is a major public health concern in low- and middle-income countries including Ethiopia. This study aimed to assess the spatiotemporal distribution of TB and identify TB risk factors in Ethiopia’s Oromia region. Descriptive and spatiotemporal analyses were conducted. Bayesian spatiotemporal modeling was used to identify covariates that accounted for variability in TB and its spatiotemporal distribution. A total of 206,278 new pulmonary TB cases were reported in the Oromia region between 2018 and 2022, with the lowest annual TB case notification (96.93 per 100,000 population) reported in 2020 (i.e., during the COVID-19 pandemic) and the highest TB case notification (106.19 per 100,000 population) reported in 2019. Substantial spatiotemporal variations in the distribution of notified TB case notifications were observed at zonal and district levels with most of the hotspot areas detected in the northern and southern parts of the region. The spatiotemporal distribution of notified TB incidence was positively associated with different ecological variables including temperature (β = 0.142; 95% credible interval (CrI): 0.070, 0.215), wind speed (β = −0.140; 95% CrI: −0.212, −0.068), health service coverage (β = 0.426; 95% CrI: 0.347, 0.505), and population density (β = 0.491; 95% CrI: 0.390, 0.594). The findings of this study indicated that preventive measures considering socio-demographic and health system factors can be targeted to high-risk areas for effective control of TB in the Oromia region. Further studies are needed to develop effective strategies for reducing the burden of TB in hotspot areas.

## 1. Introduction

Tuberculosis (TB) continues to pose a significant global health challenge despite being a preventable and treatable disease [1]. It is the leading cause of death from an infectious disease, claiming the lives of over one million people annually. The World Health Organization (WHO) reported an estimated 10 million cases of TB and 1.6 million TB-related deaths worldwide in 2021 [1]. While the magnitude of the disease varies across countries, low- and middle-income countries account for the highest burdens of the disease. Remarkably, around 87% of TB cases occur within the 30 high-TB-burden countries [2]. The African continent accounts for a quarter (25%) of the global TB cases and has the highest incidence of TB and HIV co-infection [1].

Ethiopia is one of the thirty designated high-TB-burden countries in the world, with an estimated 170,000 cases and 25,000 deaths due to TB in 2022 [1]. To combat the disease, the country has been implementing the directly observed treatment short-course (DOTS) strategy since 1994 and achieved 100% national coverage in 2006 [3]. Additionally, Ethiopia has adopted global end-TB targets aimed at reducing the TB burden. However, TB continues to be a major challenge in the country [1,4].

To accelerate progress and tailor effective interventions, it is crucial to have accurate and up-to-date information regarding areas with the highest TB burden. Previous studies conducted in Ethiopia reported spatial or spatiotemporal clustering of TB at the sub-national level and identified ecological factors associated with TB clustering [5,6,7,8,9,10]. However, these studies were limited to pre-COVID-19 pandemic data and did not assess the impact of health service coverage, including TB diagnosis services, and climatic factors on TB clustering. Furthermore, there is a lack of recent information regarding the spatial distribution and temporal patterns of TB case notifications in the Oromia region of Ethiopia.

Therefore, the objective of this study is to assess the spatiotemporal patterns in notified TB cases at the district level in the Oromia region using a Bayesian statistical framework. Additionally, this study aims to investigate the influence of environmental, socio-demographic, and health service coverage factors on the distribution of TB in the region. By showing the spatial and temporal distribution and underlying factors associated with TB distribution, this study can inform targeted interventions and guide preventive and treatment measures in the Oromia region of Ethiopia.

## 2. Materials and Methods

### 2.1. Study Setting

**Figure 1:**
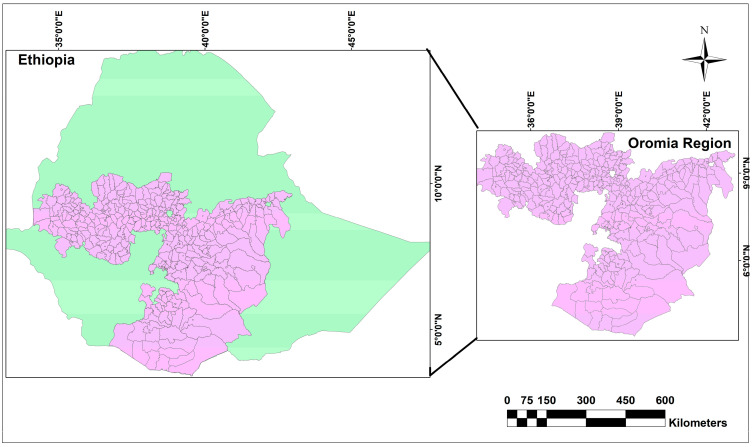
Map showing the study area, Oromia region, Ethiopia.

This study was conducted in the Oromia region of Ethiopia, one of the largest regions in the country by area with a total area of 0.2 million square kilometers (Figure 1). According to the most recent estimates, the population of the Oromia region was 40 million people in 2022 [11]. Climate conditions in the Oromia region are very diverse ranging from highland (cold), midland (temperate), and lowland (hot) areas. The climate in the region experiences four distinct seasons with hot and dry summers and mild and rainy winters. Average annual rainfall varies across the region. The districts are the lowest administrative divisions with legal autonomy.

### 2.2. Data Sources

We used different data sources for the primary outcome and exposure variables. Our primary outcome measure was the number of reported pulmonary TB cases at the district level for the period between 2018 and 2022. The TB data along with HIV data were obtained from the Health Management Information System (HMIS) [12,13] and a web-based District Health Information System (DHIS). In the HMIS and DHIS, TB cases are registered daily as the patients are diagnosed and start treatment, but they are reported quarterly in each of the health facilities that provide TB diagnosis and treatment. The data set comprises sociodemographic information on age and sex along with clinical data about the classification of TB cases [14]. Data for climatic variables such as temperature, precipitation, humidity, and wind speed were obtained from the power database [15].

Population data for the five-year study period (the number of people in each district), health service coverage, and laboratory diagnostic coverage were obtained from the Oromia Region Health Bureau (Oromia Health Bureau 2021/22 annual report). A polygon ESRI shapefile for the Oromia region at the district level was obtained from the Oromia Regional Land Bureau. The dependent variables (TB cases) were geo-referenced, and covariates were linked to the dependent variable using ArcGIS (ESRI, Redlands, CA, USA) geographical information system (GIS) software version 10.8.1.

### 2.3. Measurement

The spatial-specific population density was calculated by dividing the total number of people in each district by the land area of the district in square kilometers.

Health service coverage was calculated by dividing the number of health centers (a primary healthcare unit) by the catchment population in a specific year. The Ethiopian Ministry of Health suggests that one health center should cater to a population of 25,000 [16].

Laboratory diagnostic coverage was calculated as the proportion of health facilities providing TB diagnosis to the total number of health facilities in the district.

### 2.4. Data Analysis

TB case notification: As a descriptive analysis, the TB case notification for the Oromia region was calculated by sex and year. TB case notifications were calculated by dividing the total number of new TB cases by the population of the same year and sex in the corresponding district, multiplied by 100,000 to obtain a rate per 100,000 population.

Standardized incidence ratio: Standardized incidence ratios (SIRs) were calculated for each district by year. For each district i, i = 1…*n*, the SIR was calculated as the ratio of the observed number of TB cases in the district ($Y_{i}$) to the expected number of TB cases ($E_{i}$) in the district across the study period, *t* = 2018,…., 2022:

$$SIR_{it}\;=\;Y_{it}/E_{it}$$

The expected count $E_{it}$ represents the total number of TB cases that one would expect if the population of district i has the same risk as the regional population during the same year. The expected number of TB cases for each district ($E_{it}$) was computed as:

$$E_{it}=r_{t}^{\left(s\right)}n_{it}\;,$$

where $r_{t}^{\left(s\right)}$ is the overall crude TB case notification for the Oromia region (i.e., the total number of TB cases in a specific year divided by the total population of the region during the same year) and $n_{it}$ is the population of each district i during a year.

Spatial autocorrelation analysis: The global Moran’s I and the Getis–Ord statistics were used to identify clusters of high TB incidence at the district level across all regions of Oromia. The global Moran’s I statistic was used to assess the presence, strength, and direction of spatial autocorrelation over the Oromia region and to test the assumption of spatial independence in implementing the spatial pattern analysis. The Getis–Ord statistic was used to detect the local clustering of TB infection. Maps produced using Moran’s I statistics and the Getis–Ord statistics show the existence of TB clusters and identify the locations of potential hotspot areas.

Bayesian spatiotemporal analysis: The spatiotemporal model was constructed using covariates. The observed numbers of TB cases $Y_{i}$ in the district i for the five-year observation period were modeled using a Poisson distribution with mean $E_{it}\theta_{it}$, where $E_{it}$ is the expected number of TB cases and $\theta_{it}$ is the relative risk in the district i for a given year. The logarithm of the relative risk $\theta_{it}$ was expressed as the sum of an intercept, a vector of covariates and their coefficients, and random effects to account for extra-Poisson variability. The model for the spatiotemporal data is expressed as follows:

$$\log(Y_{it})=d_{itj}\beta_{j}+\left(\beta^{0}+\delta_{i}\right)T+\log\left(n_{it}\right)+u_{i}+v_{i}$$

where $\beta_{j}$ represents the coefficient vector of the covariates, $d_{itj}=(1,\;d_{it1},\ldots ,d_{itp})$ is the vector of p covariates corresponding to district i during a year, T corresponds to the year, $\beta^{0}$ is a measure of the significance of the regional trend in TB notification rate, $\delta_{i}$ is a measure of space–time interaction, $n_{it}$ represents the population size of districts during a year, $u_{i}$ is a random effect specific to the district i to model spatiotemporal dependence between the relative risks, and $v_{i}$ is an unstructured exchangeable component that models uncorrelated noise. The unstructured component $v_{i}$ was modeled as an independent and identically distributed normal variable with zero mean and variance $\sigma_{v}^{2}$ [17,18].

Before fitting the model, all covariates were checked for multi-collinearity using variance inflation factors (VIFs) (Table S1). Those variables with a VIF greater than 6 were excluded from the final model. Variables with a *p*-value less than 0.2 in the bivariate regression model were selected for the final model. Health service coverage, population density, temperature, precipitation, and wind speed were eligible covariates to be included in the final model. Since these independent variables had different units and scales of measurement that would have unknown threshold effects, the variables were normalized using their mean and standard deviation ([X-mean]/SD). This method also helped with identifiability in the estimation of the posterior distribution of the coefficients. All the analyses were conducted using R software version 4.3.0 and ArcGIS Pro software version 10.8.1.

## 3. Results

Table 1 shows the summary statistics for notified TB in the Oromia region reported from 337 districts to the regional TB surveillance system through the HMIS for the period January 2018 to December 2022. A total of 206,278 pulmonary TB cases were reported during the study period. Of these TB cases, 114,458 (55.5%) were males. Precisely, 51.4% (106,119) of the new pulmonary TB cases were in the age group between 15 and 34 for both sexes (Figure S1). During the study period, a total of 65,372 people tested positive for HIV; the crude HIV positivity rate of five years was found to be 0.38%. The positivity rate for HIV among tested people decreased from 0.61% in 2018 to 0.34% in 2022.

### 3.1. Case Notification

The overall TB case notification of notified TB across the study years in the Oromia region was 117.8 (95% CI 107.3–128.4) per 100,000 population. The annual TB case notification of notified TB was higher in males (ranging from 16.5 to 1201.9 per 100,000) than in females (ranging from 14.9 to 746.6 per 100,000 for females). The TB case notification of TB also varied by year, with the lowest TB case notification (96.93 per 100,000 population) reported in 2020 (i.e., during the COVID-19 pandemic) and the highest TB case notification (106.19 per 100,000 population) reported in 2019 (Table 1).

**Figure 2:**
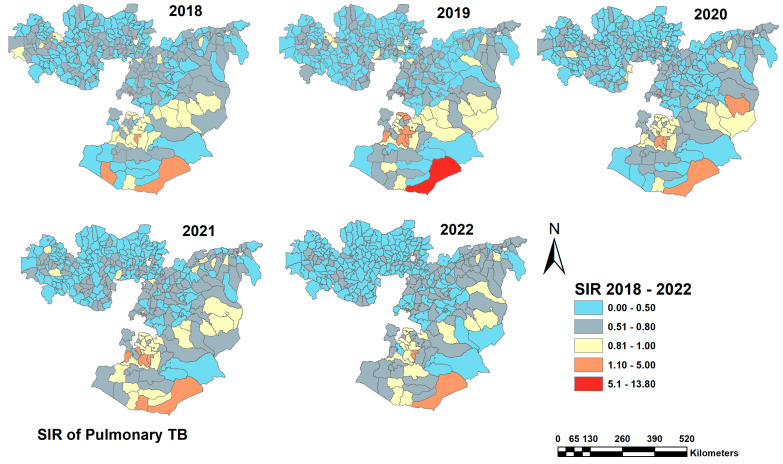
Sex- and year-standardized TB case notification (SIR) of TB cases at the district level in the Oromia region, 2018–2022. In districts with an SIR around one (color yellow), the number of TB cases observed is the same as the number of expected cases. In districts where SIR > 1 (color red), the number of TB cases observed is higher than the expected cases. Districts where SIR < 1 (color blue and gray) have fewer observed TB cases than expected.

TB cases displayed seasonal variations with the highest number of cases reported from January to March (*n* = 53,689; 26%) and the lowest number of TB reported from July to September (*n* = 49,206; 23%). The notified TB cases by year and season as a region are presented in the Supplementary Materials (Table S3). The TB case notification of notified TB varied substantially at the zonal level, ranging from 304.71 in the Horo Guduru zone to 2069.70 in Dukem town per 100,000 population. The TB case notification of notified TB at the zone level is presented in Supplementary Materials (Table S4). Spatial variation in the notified TB case notification was also observed at the district levels with a standardized incidence ratio (SIR) varying from 0.16 in Horro district in the Horro Guduru Wollega zone to 9.6 in Kercha District in the West Guji zone. Figure 2 shows the distribution of TB SIR at the district level in the Oromia region. The highest SIR of TB was found in districts located in the southern part of the region and in districts near the Somali region. The SIR of notified TB was relatively low in the western part of the region.

### 3.2. Spatial Clustering of TB

The global Moran’s index statistic value for PTB notifications in each year between 2018 and 2022 was consistently positive, ranging from 0.0657 to 0.1604 (*p*-value < 0.001). Furthermore, a significant spatial autocorrelation (Z = 5.785, *p* < 0.001) was observed in the average annual PTB notifications, indicating the presence of significantly positive spatial autocorrelation in the TB notification rate over the whole study area (Table 2). These findings suggest that the distribution of TB in the Oromia region was not random but exhibited significant spatial autocorrelation over the five years.

**Figure 3:**
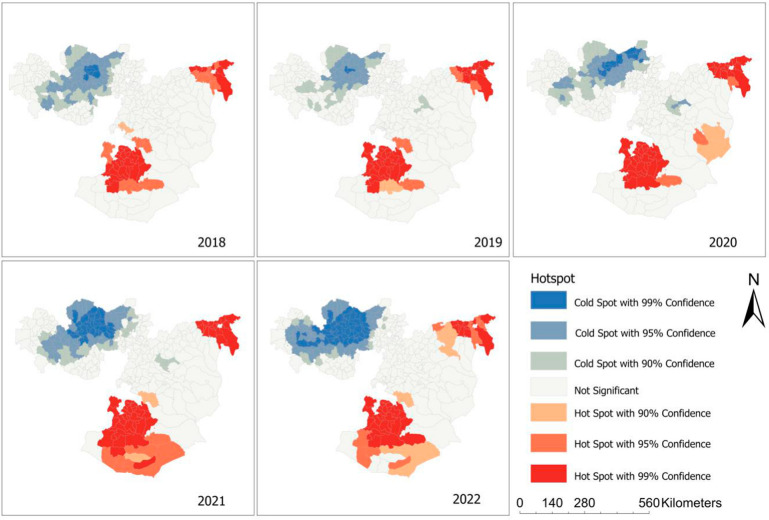
Hotspot maps showing the spatial clustering of tuberculosis incidence in the Oromia region, based on the Getis–Ord Gi* statistics, between 2018 and 2022.

Based on our clustering analyses using the Getis–Ord Gi statistic, some districts were identified as hotspots and cold spots (Figure 3).

The hotspot districts, indicating a higher-than-expected TB case notification compared with the regional average, were in the northern and southern parts of the region, while the cold spot districts were located in the western parts of the region. Over five years, the hotspots in the southern parts of the region experienced significant expansion, resulting in the spread of TB to neighboring districts.

**Figure 4:**
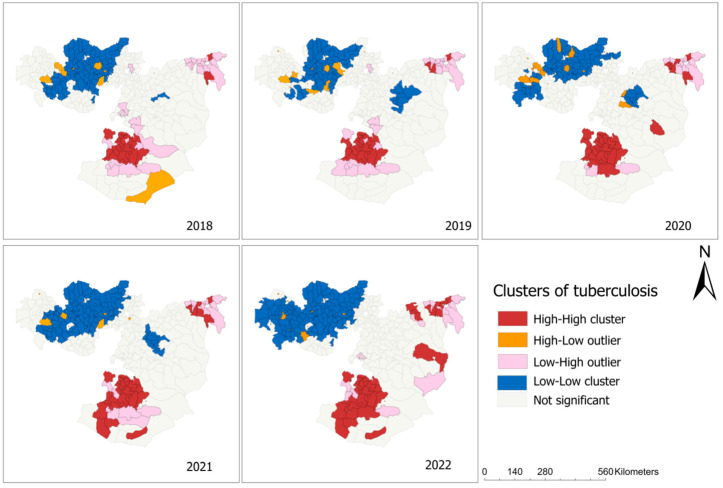
Cluster and outlier maps showing the tuberculosis incidence ratio in the Oromia region, based on Anselin Local Moran’s I analysis, between 2018 and 2022.

In the local Moran’s I analysis, districts located in the southern and northern parts of the region such as Bule Hora town in West Guji zones showed a high–high type of relationship, meaning that these districts had a high notification of TB cases and the surrounding district also had high TB case notification (Figure 3). Some districts in the western part of the region had a high–low type of relationship, indicating that there was a high TB case notification in these districts, which were surrounded by districts with a low TB case notification. Low–low clusters of TB were found in Begi districts in the West Wollega zone (Figure 4).

### 3.3. The Result from the Non-Spatial Univariate Bayesian Regression Model

According to the result of univariate analysis, all the variables examined in this study exhibited a significant association with TB notification (Table S2). However, to address the issue of multicollinearity among covariates, the variable humidity was excluded from the final model since its VIF value exceeded 6.

### 3.4. The Result of the Spatiotemporal Bayesian Regression Model

Table 3 shows the Bayesian multivariable Poisson regression model for ecological-level factors associated with TB in the Oromia region. Variables including HIV positivity rate and wind speed were negatively associated with notified TB.

**Figure 5:**
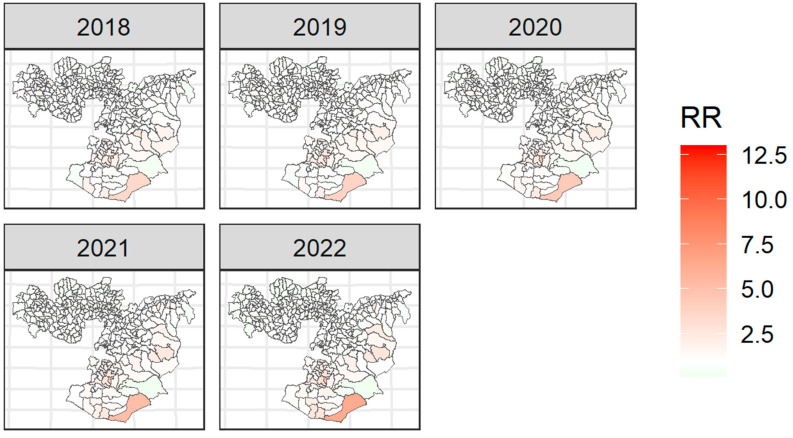
Relative risk (RR) of tuberculosis at the district level in Oromia based on the posterior spatially structured random effects, 2018-2022. The relative risk (RR) quantified whether a district has a higher (RR > 1) or lower (RR < 1) risk than the average risk in the region’s population. For example, if (RR = 2), the risk of the district is two times the average risk in the region’s population.

All variables included in the final model were significantly associated with TB incidence in the Oromia region except annual mean precipitation. After accounting for the ecological-level factors in the model, the posterior mean of spatially structured random effects was found to be clustered in the region (Figure 5). This indicates that a substantial amount of district-level heterogeneity in TB remained unexplained by the ecological level factors included in our models.

## 4. Discussion

This study conducted a comprehensive analysis of the spatiotemporal trend in TB notification within districts in the Oromia region. Its primary objectives included identifying clusters of hotspots and trends in TB case notification, evaluating the influence of socio-demographic characteristics and environmental conditions on TB prevalence, and exploring the impact of health-related factors on the distribution of the disease. By examining these factors, this study aimed to provide valuable insights into the dynamics of TB distribution within the region.

In our current study, we reported several significant findings regarding the distribution of pulmonary TB in the Oromia region. First, we observed distinct trends in TB occurrences over the study period, noting changes and patterns in both annual and seasonal occurrences. Second, we identified hotspot areas with concentrated TB cases, providing valuable insights for targeted interventions and resource allocation. Third, we identified a cluster of TB cases, indicating areas with higher transmission rates and shared risk factors. Lastly, our study revealed an association between ecological factors and TB notification, highlighting potential drivers of transmission and risk factors within the studied population in districts of the Oromia region.

*The spatial clustering of TB showed a trend in the Oromia region*. Our study also showed that TB was found to be clustered in the southern parts of the region consistently for five years. This clustering can be attributed to several factors. First, the presence of numerous mining shafts in select districts of the southern part contributes to this phenomenon. Moreover, these districts share a border with Kenyan districts as there is geographical proximity, and population movements across the border increase the likelihood of cross-border transmission of TB, which further reinforces the clustering of TB cases in this particular area. This finding emphasizes the need for coordinated efforts and strengthened collaboration between Ethiopia and neighboring countries to effectively control TB transmission in a cross-border context.

TB notification rates varied before and after the COVID-19 pandemic with the lowest TB case notification reported during the COVID-19 pandemic in 2020. Similar findings were reported in recent studies in which TB notification rates were decreased during the COVID-19 pandemic, partly due to the collapse of the health systems regarding diagnosing and reporting TB cases and due to patients’ fear of visiting health facilities [19]. As in many other countries, COVID-19 had a negative impact on TB control programs in Ethiopia, which affected TB case detection, interrupted community-based interventions, and resulted in the diversion of resources including human resources, from the TB control program to the COVID-19 response [1,20].

We observed notable seasonal variations in the notification of TB; specifically, our findings indicated a peak in TB cases between January and March (dry season) with a trough from July to September (rainy season). The findings in this study corroborate with a study conducted in Ethiopia, which showed higher and lower TB case notifications during the dry and rainy seasons, respectively [21,22,23,24]. In Ethiopia, the dry season occurs from January to March, immediately following the harvest season in most rural societies. During these periods, agricultural activities become less demanding, allowing individuals in rural societies to experience the freedom to visit health facilities, and the probability of TB diagnosis during this time frame will be high. Conversely, the rainy season, specifically from July to September is characterized by intensive agricultural activities among the rural population, whose livelihoods are heavily dependent on farming. In addition, during these months, individuals in rural communities dedicate extensive hours to working in their fields, while the healthcare-seeking tendency is restricted due to heavy rainfall and agricultural commitments. These situations could potentially contribute to a decreased number of TB cases. These findings suggest the need to investigate season-specific strategies for TB case finding in Ethiopia.

*Densely populated districts were highly vulnerable to TB distribution.* The spatiotemporal distribution of TB was positively associated with demographic factors. Accordingly, our present study revealed an association between TB notification and population density. This aligns with previous studies that consistently indicate a relatively higher prevalence of TB in areas characterized by higher population density, particularly in urban settings, when compared to less densely populated rural areas [10,25,26,27]. In areas with high population density, where overcrowded living conditions are common, there is an increased risk of TB transmission due to the proximity of individuals [28]. Moreover, the limited access to quality healthcare services and poor sanitation exacerbate the situation. The combination of these factors, along with a higher prevalence of HIV and other risk factors, contributes to the occurrence of high TB prevalence [4,10]. By addressing the unique challenges posed by population density, health authorities can work toward reducing the prevalence of TB in these areas and improving overall health outcomes.

Our study also showed that TB case notification was higher in males than females. Similar studies conducted in different parts of the world including Ethiopia revealed that a higher TB case notification of TB was observed in males than in females [7,9,29,30]. This might be attributed to their strong social interactions, staying in overcrowded situations, and the high drinking and smoking habits of males, all of which contribute to the development of TB [31,32]. Immunological differences between males and females (which favors women) and the impact of sex hormones on TB susceptibility and progression [33] might also contribute to the occurrence of higher TB in males. On the other hand, health-seeking behavior, stigma, socioeconomic determinants and barriers, and misdiagnosis (such as poor quality of TB screening during pregnancy) [32], might be associated with lower TB case notification of notified TB in females. Efforts aimed at mitigating gender disparities in TB incidence should prioritize the promotion of gender equity, enhancing healthcare accessibility, and fostering disease awareness among individuals of all genders.

*Meteorological factors should be considered in TB control and prevention strategies.* Our current study revealed a significant correlation between meteorological factors and the notification of TB cases. Specifically, we found a positive relationship between temperature and TB case notification. These findings are consistence with many studies conducted globally, including Ethiopia, which also reported a link between temperature and TB notification, particularly in the case of pulmonary TB [23,34,35,36,37]. The association between temperature and TB notification has been widely acknowledged and assumed in the scientific community. However, extremely high or low temperatures can elevate the likelihood of growth and transmission of *M. tuberculosis* which is the causative agent for TB [38,39]. Thus, our study further reinforces the understanding that temperature plays a crucial role in influencing the occurrence and distribution of TB cases.

Additionally, in our present study, the variable wind speed was found to have the opposite effect on TB notifications. A similar finding was reported in a study conducted in mainland China [40,41]. The transmission of *M. tuberculosis* may be hindered by low wind speed [42,43,44]. Further explorations are necessary to fully comprehend the mechanism underlying the negative correlation observed between wind speed and TB distribution.

### District-Level Primary Health Care Service Strongly Affected TB Case Notification

In our present study, district-level health service coverage was positively associated with TB case notification. Many studies revealed a direct association between TB case notification and health service coverage. Based on this, increased TB case notification can be justified by improved health service coverage [28,36,45]. As part of health service coverage, community-level health extension workers carry out the identification of TB suspects and refer the suspects for diagnosis, which might lead to higher levels of case identification, resulting in increased TB case notification [46]. Enhanced district-level health service coverage and investing in community-level health extension workers can improve TB case notification.

As part of health service coverage, increased TB case notification can be justified with laboratory diagnostic coverage [47]. In our present study, laboratory diagnostic coverage is positively associated with TB case notification. Many studies revealed the direct relationship between TB notification and laboratory diagnostic coverage or access. When there is wider access to laboratory diagnostic services for TB, more cases can be accurately diagnosed and confirmed. This leads to an increased number of TB cases being notified to the health system.

The variable HIV positivity ratio was found to be negatively associated with TB case notification in our current study. This type of finding is not common as there is a direct relationship between HIV and TB [1,48,49,50]. The negative association observed in this study may be attributed to several factors, including the provision of TB preventive therapy, the prompt or the same-day initiation of antiretroviral therapy (ART), and clinical evaluation for individuals diagnosed with HIV. These interventions, such as rapid ART initiation and the same-day service along with strong TB/HIV collaborative activities, potentially play a significant role in influencing the observed negative association. Further studies are needed to identify additional factors that could contribute to the observed negative association to gain a more comprehensive understanding of the phenomenon.

This study made significant contributions to the spatiotemporal analysis of TB case notification in the Oromia region of Ethiopia. All districts in the region were included in the analysis. However, there are some limitations. Firstly, the data used in this study were extracted from a centralized HMIS. This raises the possibility of under-reporting TB cases in some districts. In other words, the low TB case notification rates in many districts may not accurately reflect the actual burden of the disease in those districts as the extrapulmonary TB case category was not included in the analysis. A wide range of data was used as independent variables, sourced from different outlets, such as -non-HMIS or parallel reports and websites, which were compiled by different organizations. Consequently, the analysis of the relationship between data from these diverse sources may introduce certain biases that can impact the obtained results. Another limitation of this study is that age-specific analysis was not performed.

## 5. Conclusions

Substantial spatial variations in the distribution of notified TB case notifications were observed at zonal and district levels with most of the hotspot areas detected in the northern and southern parts of the region. The spatiotemporal distribution of notified TB incidence was positively associated with population density. Efforts aimed at combating TB should extend beyond simply halting its transmission. It is important to proactively assess the potential distribution of diseases by analyzing past occurrences concerning diverse ecological factors. Implementing preventive measures that account for socio-demographic, meteorological, and health system factors can be strategically focused on areas at high risk, resulting in effective control in the Oromia region.

## Figures and Tables

**Table 1:** TB and HIV case notification per 100,000 population in the Oromia region of Ethiopia, 2018–2022.

Years	TB Case Notification per 100,000 Population ^			HIV Case Notification per 100,000 Population *
	Male	Female	Total	Total
2018	111.63	94.66	103.21	608.33
2019	118.01	94.20	106.19	373.68
2020	105.92	87.80	96.93	278.24
2021	116.65	94.16	105.49	344.18
2022	111.91	88.90	100.49	343.19
Five years	564.10	459.38	512.13	

^ TB case notification was calculated by dividing the total number of TB reported in the region by the total population in the region; * HIV case notification was calculated by dividing the total number of HIV-positive individuals by the total number of people tested for HIV.

**Table 2:** Global spatial autocorrelation in the TB notification rate in Oromia region, Ethiopia, 2018–2022.

Year	Moran’s	Z-Score	*p*-Value	Pattern
2018	0.084146	3.995610	<0.001	Clustered
2019	0.065740	3.217874	0.0013	Clustered
2020	0.089804	4.292228	<0.001	Clustered
2021	0.155662	7.454590	<0.001	Clustered
2022	0.160383	7.759326	<0.001	Clustered
Annual Average	0.121247	5.785267	<0.001	Clustered

**Table 3:** Bayesian spatiotemporal Poisson regression model with spatially random effects for notified incidence of TB in the Oromia region of Ethiopia, 2018–2022.

Independent Variables	Coefficient (95%CrI)
Year	−0.058 (−0.071, −0.046)
HIV Positivity Rate	−0.012 (−0.017, −0.006)
Population density	0.491 (0.390, 0.594)
Laboratory diagnostic coverage	0.090 (0.014, 0.166)
Health Service coverage	0.426 (0.347, 0.505)
Precipitation	0.015 (−0.009, 0.038)
Temperature	0.142 (0.070, 0.215)
Temperature (squared term)	0.066 (0.037, 0.096)
Wind Speed	−0.140 (−0.212, −0.068)
DIC	16,054.71

## Data Availability

The information presented in this study is available on request from the corresponding author.

## Supplementary Materials

The following supporting information can be downloaded at: https://www.mdpi.com/article/10.3390/tropicalmed8090437/s1, Table S1: Variance inflation factors; Table S2: Univariate model for notification of TB in the Oromia region of Ethiopia, 2018–2022; Table S3: Yearly and quarterly (seasonal) notified TB cases by sex; Table S4: The incidence sate of tuberculosis in the Oromia region at the zone level, between 2018 and 2022.; Figure S1: Age–Sex pulmonary TB distribution.

## Abbreviation

CrI	credible interval
DIC	Devian Information Criteria
DHIS	District Health Information System
DOTS	Directly Observed Treatment Short Course
GIS	geographical information system
HIV	human immunodeficiency virus
HMIS	Health Management Information System
RR	relative risk
SIR	standardized incidence ratio
SD	standard deviation
TB	tuberculosis
VIF	variance inflation factors
WHO	World Health Organization
