# Supplementary material for: Spatiotemporal Distribution of Tuberculosis in the Oromia Region of Ethiopia: A Hotspot Analysis
Source: Trop Med Infect Dis. 2023 Sep 7;8(9):437. doi: 10.3390/tropicalmed8090437 (PMC10536582; doi:10.3390/tropicalmed8090437)
Supplement: Supplementary file 1 [file tropicalmed-08-00437-s001.zip › tropicalmed-2555888-supplememtary.pdf]

Tropical Medicine and Infectious Disease

Supplementary Materials: Spatiotemporal distribution of tuberculosis in the Oromia re-gion of Ethiopia: Hotspot analysis

Dereje Bekele[1,2], Solomon Aragie[2], Kefyalew Addis Alene[3], Tariku Dejene[4], Samson Warkaye[5], Melat Mezemir[6], Dereje Abdena[1], Tesfaye Kebebew[7], Abera Botore[7], Geremew Mekonen[1], Gadissa Gutema[2,8], Boja Dufera[2,9], Kolato Gemede[1], Birhanu Kenate[7], Dabesa Gobena[7], Bizuneh Alemu[1], Dagnachew Hailemariam[1], Daba Muleta[7], Gilman SIU[10], Ketema Tafess[11,12]

Table S1. Variance inflation factors.

Variables	VIF
HIV Positivity Rate	1.095
Population density	1.210
Laboratory diagnostic coverage	1.216
Health Service coverage	1.069
Precipitation	5.269
Temperature	3.492
Wind Speed	2.034
Year	1.001
Humidity	8.978

* Variables with VIF greater than six and above were excluded from the model.

Table S2. Univariate model for notification of TB in the Oromia region of Ethiopia, 2018–2022.

Independent variables in univariate Poisson regression*	TB Notification	
	Coefficient	P-value
HIV Positivity Rate	0.075896	< 0.001
Population density	0.312771	< 0.001
Laboratory diagnostic coverage	0.056826	< 0.001
Health Service coverage	0.414084	< 0.001
Precipitation	-0.026815	< 0.001
Humidity	0.023932	< 0.001
Temperature	0.068449	< 0.001
Wind Speed	0.090209	< 0.001
Year	-0.033156	< 0.001

* For all univariate models, “*year*” was considered as a control variable.

Table S3. Yearly and Quarterly (Seasonal) Notified TB Cases by Sex.

Year	Quarter/Season	Male	Female	Total TB Case
2018	January - March	5,040	4,437	9,477
	April - June	5,174	4,266	9,440
	July - September	5,381	4,285	9,666
	October - December	5,785	4,873	10,658
2019	January - March	5,819	4,467	10,286
	April - June	5,845	4,610	10,455
	July - September	5,704	4,332	10,036
	October - December	5,884	4,874	10,758
2020	January - March	5,851	4,959	10,810
	April - June	4,433	3,565	7,998
	July - September	5,150	4,099	9,249
	October - December	6,035	4,908	10,943
2021	January - March	5,907	4,761	10,668
	April - June	5,937	4,705	10,642
	July - September	6,132	4,825	10,957
	October - December	6,348	5,050	11,398
2022	January - March	6,856	5,592	12,448
	April - June	6,918	5,409	12,327
	July - September	5,285	4,013	9,298
	October - December	4,974	3,790	8,764
2018 to 2022	January - March	28,307	22,555	50,862
	April - June	29,473	24,216	53,689
	July - September	27,652	21,554	49,206
	October - December	29,026	23,495	52,521

Table S4. The incidence rate of tuberculosis in the Oromia region at the zone level, between 2018 and 2022.

Zone/Town	TB incidence rate per 100,000 population					
	2018	2019	2020	2021	2022	Total
Adama Town	103.64	141.59	96.88	111.72	135.48	589.31
Ambo Town	124.83	175.27	114.06	149.61	207.53	771.30
Arsi	103.05	96.88	83.13	88.61	92.67	464.33
Asela Town	126.77	132.98	119.30	96.89	160.63	636.57
Bale	124.30	119.79	97.85	111.67	110.50	564.11
Batu Town	167.55	136.45	105.24	97.65	159.44	666.33
Bishan Guracha Town	214.02	70.96	46.61	55.05	89.17	475.82
Bishoftu Town	108.79	104.99	87.30	96.20	106.24	503.52
Borena	119.45	153.22	136.64	214.53	225.63	849.46
Buno Bedele	79.10	87.21	97.85	87.96	73.27	425.39
Burayu Town	146.45	133.27	99.00	118.57	153.36	650.64
Dukem Town	262.82	497.30	470.39	424.94	414.25	2069.70
East Bale	114.16	117.85	119.53	143.19	153.28	648.01
East Harerge	102.40	113.20	103.44	122.33	119.30	560.67
East Shewa	104.21	102.71	92.77	93.78	83.63	477.10
East Wollega	79.15	82.17	76.07	59.95	43.47	340.80
Finfinne Surrounding	99.61	107.49	82.92	84.11	89.44	463.56
Gelan Town	72.92	146.34	85.50	64.48	95.65	464.88
Guji	187.75	195.93	182.90	202.91	226.38	995.87
Holeta Town	112.74	150.18	103.87	105.80	167.95	640.54
Horo Guduru Wollega	66.02	75.14	65.02	55.29	43.25	304.71
Ilubabor	79.43	92.91	95.95	88.81	59.77	416.86
Jimma	77.41	85.08	84.50	84.64	73.61	405.24
Jimma Town	149.06	181.26	145.68	179.01	194.90	849.91
Kelem Wollega	117.11	106.34	98.58	96.64	74.40	493.07
Lege Tafo Town	215.25	211.00	177.21	190.34	174.92	968.71
Mojo Town	186.49	148.89	112.40	120.21	171.60	739.59
Nekemte Town	123.90	121.26	73.86	88.08	115.47	522.57
North Shewa	72.03	72.25	51.52	60.54	51.84	308.19
Robe Town	109.80	108.02	107.48	75.25	138.62	539.17
Sebeta Town	73.16	77.25	62.91	52.84	73.48	339.64
Shashemene Town	292.28	261.33	263.12	241.29	305.71	1363.74
South West Shewa	95.21	99.16	89.17	93.84	70.33	447.71
Sululta Town	269.18	224.67	158.14	262.18	284.76	1198.93
Weliso Town	122.22	208.38	192.60	129.41	133.76	786.37
West Arsi	107.77	101.71	86.38	97.58	89.26	482.70
West Guji	165.71	179.59	196.60	234.11	201.39	977.40
West Harerge	100.82	94.02	106.29	128.46	143.39	572.99
West Shewa	77.73	81.06	73.16	69.60	52.45	353.99
West Wollega	89.29	91.04	75.35	78.03	54.11	387.83
Oromia	103.21	106.19	96.93	105.49	100.49	512.13
