# Supplementary figures and images for: Spatiotemporal Distribution of Tuberculosis in the Oromia Region of Ethiopia: A Hotspot Analysis
Source: Trop Med Infect Dis. 2023 Sep 7;8(9):437. doi: 10.3390/tropicalmed8090437 (PMC10536582; doi:10.3390/tropicalmed8090437)

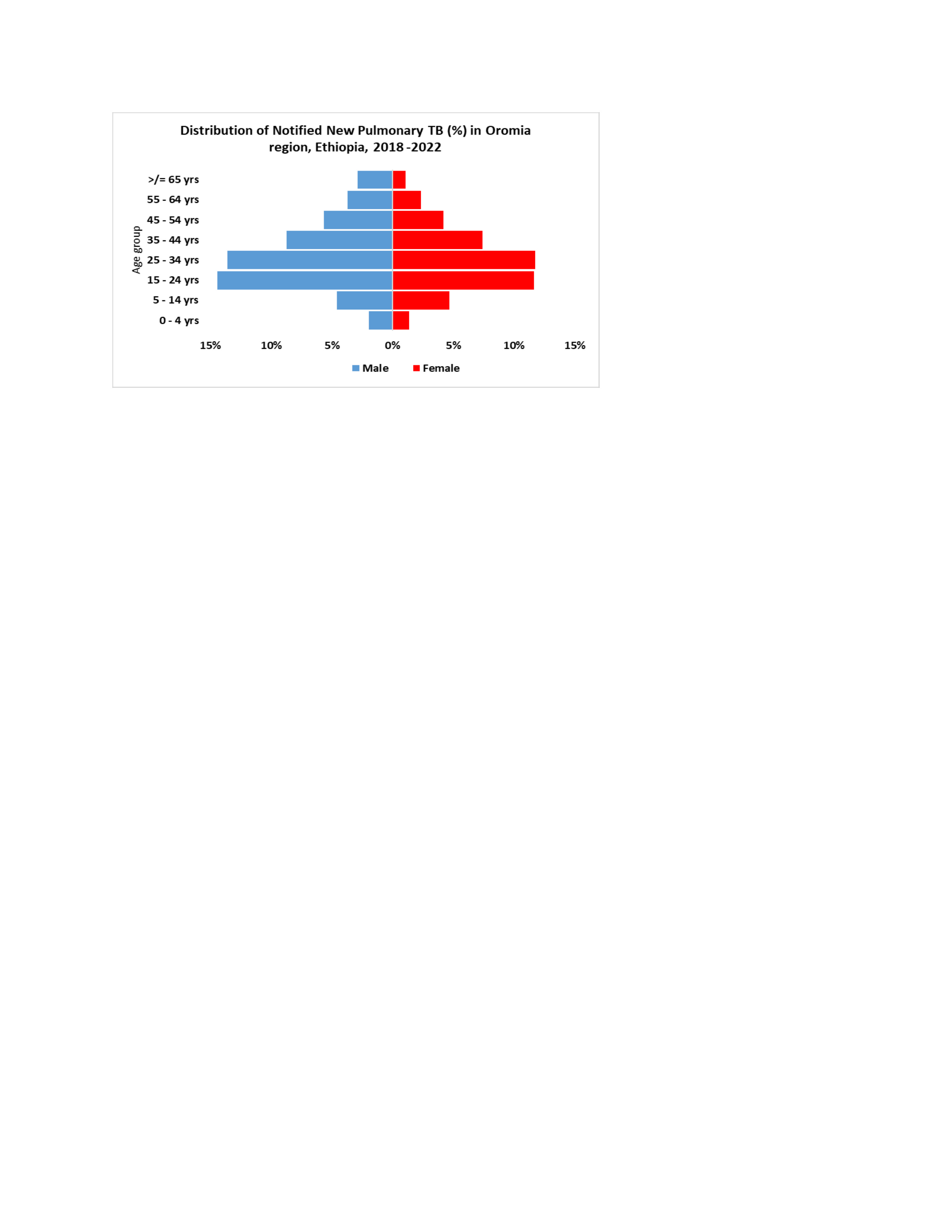

Supplement: Supplementary file 1 [file tropicalmed-08-00437-s001.zip › Figure S1 Age sex PTB distribution.tif]
